# Supplementary material for: LmCht5-1 and LmCht5-2 Promote the Degradation of Serosal and Pro-Nymphal Cuticles during Locust Embryonic Development
Source: Biology (Basel). 2022 Dec 7;11(12):1778. doi: 10.3390/biology11121778 (PMC9775170; doi:10.3390/biology11121778)
Supplement: Supplementary file 1 [file biology-11-01778-s001.zip › biology-2044090-supplementary.pdf]

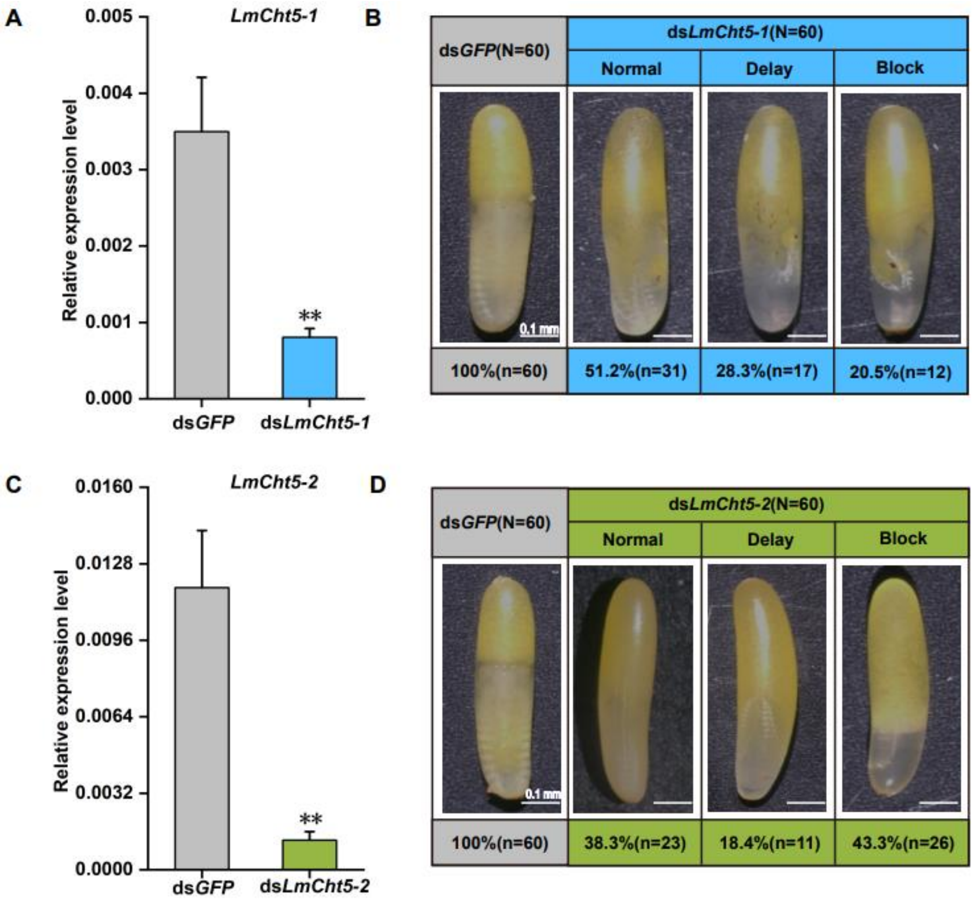

Figure S1. Microinjection of ds*LmCht5-1* and ds*LmCht5-2* block locust development during the early serosal cuticle degradation A and C. The mRNA expression level of *LmCht5-1* (A) and *LmCht5-2* (C) detected by RT-qPCR is reduced at E6 after microinjection of ds*LmCht5-1* and ds*LmCht5-2* at E5, respectively. B and D, Katatrepsis is the typical development of locust egg during E5 to E7. Microinjection of ds*LmCht5-1* (B) and ds*LmCht5-2* (D) at E5 caused the blocking and delay of katatrepsis in certain extent, respectively. Scale bar is 0.1 mm.

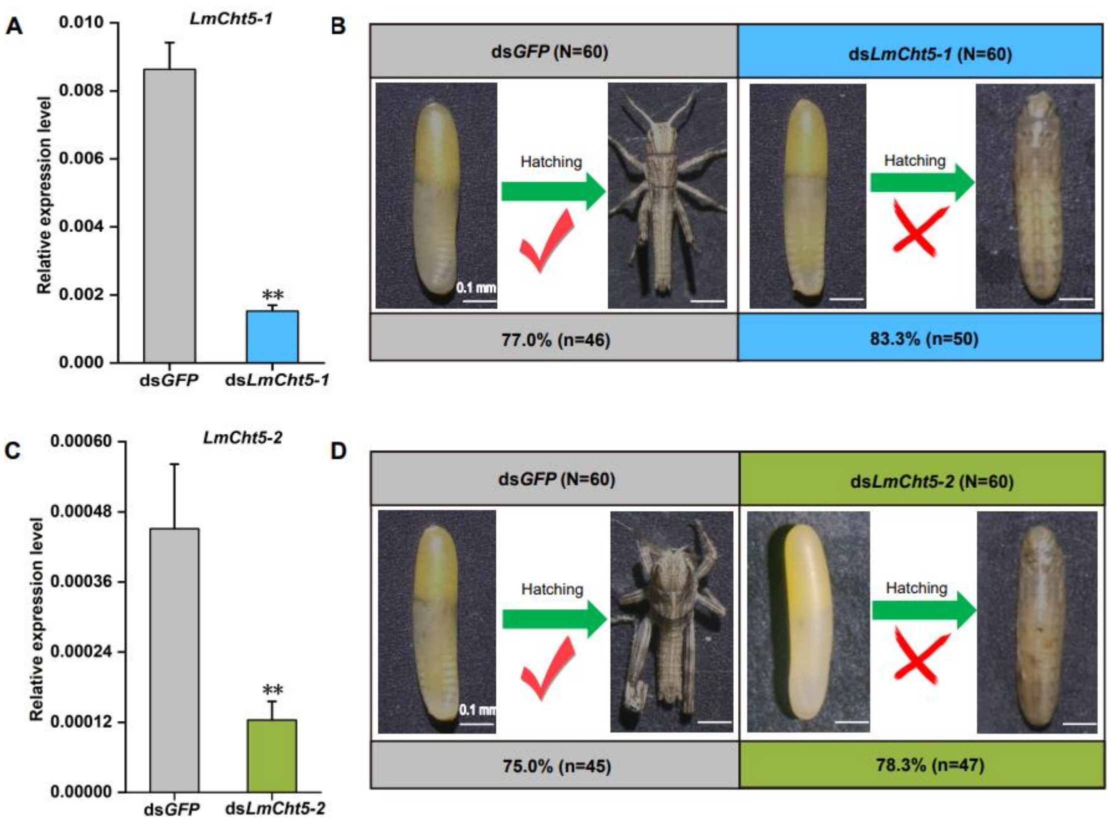

Figure S2. Microinjection of ds*LmCht5-1* and ds*LmCht5-2* block locust hatching. A and C. The mRNA expression level of *LmCht5-1* (A) and *LmCht5-2* (C) detected by RT-qPCR is reduced at E8 after microinjection of ds*LmCht5-1* and ds*LmCht5-2* at E7, respectively. B and D. Hatching from egg is the typical developmental phenotype of locust egg at E13 or E14. Microinjection of both ds*LmCht5-1* (B) and ds*LmCht5-2* (D) at E14 blocked hatching rate. Scale bar is 0.1 mm.
